# The Scientific Impact of Developing Nations

**DOI:** 10.1371/journal.pone.0151328

**Published:** 2016-03-29

**Authors:** Claudia N. Gonzalez-Brambila, Leonardo Reyes-Gonzalez, Francisco Veloso, Miguel Angel Perez-Angón

**Affiliations:** 1 Business School, Instituto Tecnologico Autonomo de Mexico, Mexico City, Mexico; 2 Católica Lisbon School of Business and Economics, Universidade Católica Portuguesa, Lisbon, Portugal; 3 Physics Department, Centro de Investigación y de Estudios Avanzados del IPN, Mexico City, México; Universidade de Brasília, BRAZIL

## Abstract

This paper analyzes science productivity for nine developing countries. Results show that these nations are reducing their science gap, with R&D investments and scientific impact growing at more than double the rate of the developed world. But this “catching up” hides a very uneven picture among these nations, especially on what they are able to generate in terms of impact and output relative to their levels of investment and available resources. Moreover, unlike what one might expect, it is clear that the size of the nations and the relative scale of their R&D investments are not the key drivers of efficiency.

## Introduction

Perhaps the most widely used tool to judge a nation’s scientific performance is international comparison with peer countries [1–3]. This research typically compares publications and citations that the scientific work of each country receives, to look at relative impact. It also analyzes efficiency, comparing countries on indicators such as papers or citations per level of expenditures. Despite these important studies, an important limitation is that they mostly focus on developed countries. But looking at the scientific performance of developing nations is at least as relevant.

First, developing countries typically finance most of their research with public funds and face stronger budget austerity, making it more important to understand the effect of Research and Development (R&D) budget allocation decisions. Second, over the last few years, an increasing number of developing nations, including Brazil, China, India and Mexico, have proclaimed their commitment to science and technology (S&T) as a fundamental pillar of their economic development. Finally, there is an increasing debate on perceived performance of the national science systems of these countries, but hardly any quantitative analysis to support the discussion.

In this article we characterize and compare the investment and performance of S&T for a group of developing countries. We focus our analysis on nine nations: Argentina, Brazil, Chile, China, India, Mexico, Poland, South Africa and Turkey (which will be named the baseline group). These countries were chosen because they are among the largest developing nations, have heterogeneous S&T dynamics, there is sufficient data available to construct all the relevant benchmarks, and their individual contribution to the global S&T, measured both as share in R&D investment and scientific output, was higher than 0.1% of the world total in the 2000–2009 period.

Similar to [3], we consider R&D investment as a measure of inputs and the number of citations as a measure of scientific impact. We then assess efficiency, considering as a measure of input the R&D investment and a number of productivity indicators, from publications and citations per researcher (the same measure used by [3]) to patents and citations per unit of Gross Domestic Product (GDP), as measures of outcomes. [3] uses citations per unit of GDP as a measure of output/outcome. Similarly, we include the number of patents per unit of GDP as a measure of outcome of a S&T system [4]. The results are contrasted against those for a set of benchmark developed nations, including Spain, South Korea, the United States (US), the 15 European Union nations (EU15 include Austria, Belgium, Denmark, Finland, France, Germany, Greece, Ireland, Italy, Luxembourg, the Netherlands, Portugal, Spain, Sweden and the United Kingdom) and the Russian Federation, extending and complementing the work by [3]. We include Spain and South Korea because these nations have improved significantly in economic and scientific terms in the last two decades and, like most developing nations, they also face language barriers when trying to publish in international (mostly English) peer reviewed journals. The United States, the European Union (EU15) and the Russian Federation served as general benchmarks.

### Widening Gap?

The first critical issue is to examine what is happening to science in the developing world compared with the developed world. According to [3] “the nations with the most citations are pulling away from the rest of the world.” This conclusion, however, may be the product of emphasizing the context of advanced nations, treating the developed world as just a block. A more detailed analysis reveals a different and richer portrait.

Between 1993 and 2009, the total world investment on R&D grew from approximately USD 452 to USD 1,276.9 billions [5]; an average growth of 6.7% each year (current Purchasing Power Parity—PPP values). In the same period, our baseline group of developing nations grew in absolute terms at almost double that rate (12.4%) almost tripling their level of expenses during this period. This increase is even larger in China, Turkey and Mexico, which increased their R&D investment at an average rate of 18.8%, 12.6% and 10.6%, respectively; these increments contrast especially with the US and the EU15, who had their expenditure growing much less (5.7% and 5.2%, respectively).

A similar perspective exists for outputs. World scientific output, measured as the number of publications in the Web of Science [6], grew 4.2% each year, going from 682,064 in 1996 to 1,164,000 papers in 2009. In the same period, the baseline group once again grew at more than three times that rate (13%). China and Turkey grew at a higher rate than the world (17% and 16% respectively), while the EU15 grew at almost the world pace (3.5%) and the US and the Russian Federation had lower rates (2.3% and 0.2%, respectively).

In addition to absolute growth rates, we also want to understand how countries are contributing relative to others and to the world total. Thus, we calculated each country’s share in total world investment and scientific impact. These are represented in Table 1. We used citation share instead of publication share because citations provide a better measure of quality and visibility of the results of science [7–8]. Then, we calculated the growth rates of these shares for the period 1993–2009 for GERD and 1991–2009 for citation share in five-year periods. The GERD share is the contribution of each country to the total investment in the world. For example, the United States invested 28.7% of the total investment in R&D around the world in 2009. The citation share refers to the proportion of cites that a given country received relative to the total number of citations that were generated around the world in a given period.

**Table 1:** GERD and Citation share, and share’s annual growth rate.

	GERD share (%)				Citation share (%)			
Country	1993	2001	2009	Share’s annual growth rate*	1991–1995	1999–2003	2005–2009	Share’s annual growth rate*
Argentina	0.40%	0.20%	0.30%	-1.78%	0.10%	0.30%	0.31%	6.49%
Brazil	1.80%	1.80%	1.90%	0.34%	0.30%	0.60%	0.95%	6.61%
Chile	0.10%	0.10%	0.10%	0.00%	0.10%	0.20%	0.21%	4.21%
China	3.60%	7.40%	12.10%	7.87%	0.50%	1.50%	3.59%	11.57%
India	2.50%	3.30%	2.10%	-1.08%	0.50%	0.80%	1.21%	5.03%
Mexico	0.30%	0.50%	0.50%	3.24%	0.10%	0.30%	0.36%	7.38%
Poland	0.40%	0.30%	0.30%	-1.78%	0.40%	0.60%	0.73%	3.40%
Turkey	0.30%	0.40%	0.70%	5.44%	0.10%	0.30%	0.57%	10.15%
South Africa	0.40%	0.40%	0.40%	0.00%	0.20%	0.30%	0.32%	2.65%
**Baseline Group**	**9.40%**	**14.00%**	**18.40%**	**4.29%**	**2.40%**	**4.80%**	**8.25%**	**7.10%**
South Korea	2.20%	2.70%	3.50%	2.94%	0.20%	0.90%	1.44%	11.59%
Spain	1.10%	1.10%	1.35%	1.29%	1.20%	2.20%	2.47%	4.09%
Russian Feder.	1.70%	1.70%	2.60%	2.69%	0.40%	1.10%	0.83%	4.14%
USA	36.80%	35.50%	28.70%	-1.54%	44.20%	39.00%	29.35%	-2.25%
EU15	28.00%	24.30%	19.50%	-2.24%	26.80%	30.80%	21.05%	-1.33%

* Compound Annual Growth Rate.

Sources: GERD share data was obtained from OECD Main Science and Technology Indicators (http://stats.oecd.org/Index.aspx?DataSetCode=MSTI_PUB), and UNESCO (2010 and 2013).

Citation share was calculated using data of the ISI National Science Indicators (Thomson Reuters, 2011).

Table 1 suggests that, by 2009, two major groups emerge: one group of mid-level contributors includes Brazil, India, and the Russian Federation. By contrast, Argentina, Chile, Mexico, Poland, South Africa and Turkey are smaller contributors, remaining below 0.7% of the world total in terms of investment and impact. In addition, over the 18 year period considered in the analysis, China, South Korea and Spain departed from the mid-level group and became much greater contributors to global S&T.

We can then compare and contrast individual countries on how they are contributing to world S&T on outputs/impacts, given their contribution on the input side. First, it is important to single out, in 2009, Argentina, Chile, Spain, and specially Poland, are countries that are having a relatively higher impact contribution in comparison to what they are investing. This is, their citation share, in the 2005–2009 period, was higher than their GERD share in 2009. This suggests an efficient use of their resources in terms of their overall contribution to the international pool of scientific knowledge. At the same time, a number of other nations invest as much, and often more as any of the four highlighted above, but achieve inferior levels of relative impact (e.g. Brazil and the Russian Federation).

The table also provides a temporal evolution. In the 1993–2009 period, Brazil, Chile and South Africa became relatively more efficient, because they increased their citation share while maintaining their share of investment almost constant; also Mexico, China and Turkey increased both shares but the increase in the citation share was even larger; South Korea is the most efficient country, as its citation share grew almost three times its investment share. In contrast Argentina, India and Poland reduced their investment share while increasing their contribution to the world science.

The significant growth trend for these nations, contrasts with those of the US and the EU15. The share of world R&D investment for the baseline group was growing at an annual rate of 4.29% during the period noted in the table, and its citation share grew at 7.1% per five-year period. In the same eighteen years, the US saw its share of world investment in R&D reduced at an annual rate of 1.54%, while the EU15 shrank at 2.24% per year. On the impact side, the US had a decline of 2.25% per year in citations, while the share of the EU15 citations grew at an annualized rate of 1.33%. Overall, these differences in growth rates support the notion that this important group of developing countries is catching up with the developed ones.

### Inputs and outputs/outcomes for R&D

To analyze efficiency, we use a modified version of the superimposed footprint graph introduced by [3]. This tool gives a “snapshot of value-for-money measured by the international impact of the research output of each nation”, allowing a comparison of the productivity of different systems. Following the approach of [3], the data of each country was averaged over several years, 2005–2009 was the period we used.

Two measures of input were considered. First, the most important overall indicator, the gross domestic expenditure on R&D (GERD) as a percentage of GDP, which consists of the sum of all annual investments on R&D in business, university, government and not-for-profit sectors, expressed as a percentage of GDP of the national territory. A second measure, the business enterprise expenditure on R&D (BERD) as percentage of GDP, was included to show the contribution of the private sector in the total expenditure. This measure was also expressed as a percentage of GDP of the national territory. To measure the scientific productivity of each nation, and considering the important differences in the sizes of economies and the scientific communities we use the number of publications and citations per researcher and the number of resident patents and citations per unit of GDP.

**Fig 1:**
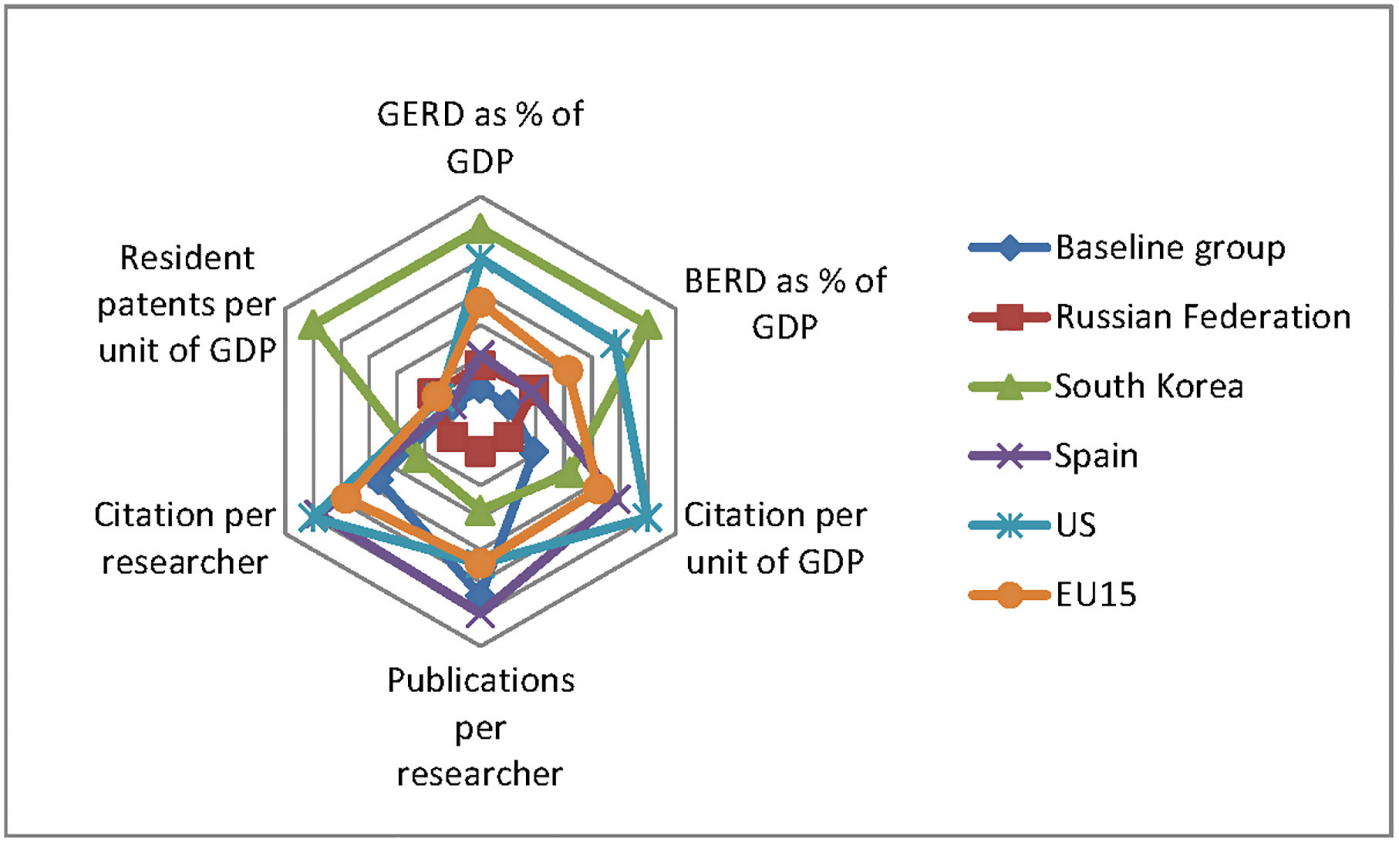
Input-Output for R&D baseline group vs. developed economies. Sources: GERD, BERD and the number of full time equivalent (FTE) researchers data were obtained using the OECD Main Science and Technology Indicators (http://stats.oecd.org/Index.aspx?DataSetCode=MSTI_PUB). The number of publications and citations was obtained using the ISI National Science Indicators (Thomson Reuters, 2011). The GDP was obtained from the World Bank Indicators www.worldbank.org. Patents were obtained from WIPO, *Industrial Property Statistics—Aggregate Patent Data*, (2013).http://www.wipo.int/ipstats/en/statistics/patents/index.html.

In Fig 1 the data of each country is the average over the 2005–2009 five year period. All data was normalized to the maximum value of each indicator. The purpose of using radar charts was to examine the relative values of the different variables. Thus, the data length is proportional to the maximum magnitude of each indicator. Each star represents a country.

Fig 1 compares the average performance of the baseline group against Spain, South Korea, the US and the EU15. As expected, the more developed countries outperform the baseline group in almost all dimensions but publications per researcher. This could be a consequence that most developing countries’ S&T institutions were inspired by what is known as the “linear model”, assuming that good-quality basic research could produce, eventually, applied research that would increase the society welfare.

On the input side, it is important to note that the gap between the baseline group and the rest of the nations is more important on the business side of expenditures than in overall expenditures. This shows that public expenditures play a disproportionately larger role in the meager investments that these nations do in S&T when compared to the developed world. Moreover, when looking at the output/outcome side it is also clear that the countries in our baseline group trail the developed nations also in the level of efficiency with which they use their scarcer resources, having lower citations per researcher, but higher publications per researcher. Yet, it is interesting to note that the baseline group performs better than South Korea in terms of publication and citation indicators, suggesting that magnitude of the investment is far from being the only explanation for the differences. The graph also suggests that the commitment of business to R&D is reflected in the overall orientation of the national system and in the metrics where it leads or trails. South Korea, where the business community is heavily involved in R&D funding, is where the contrast in terms of science and invention productivity is more striking. It is behind in all science productivity measures, but it leads in its ability to generate patents given its level of development.

**Fig 2:**
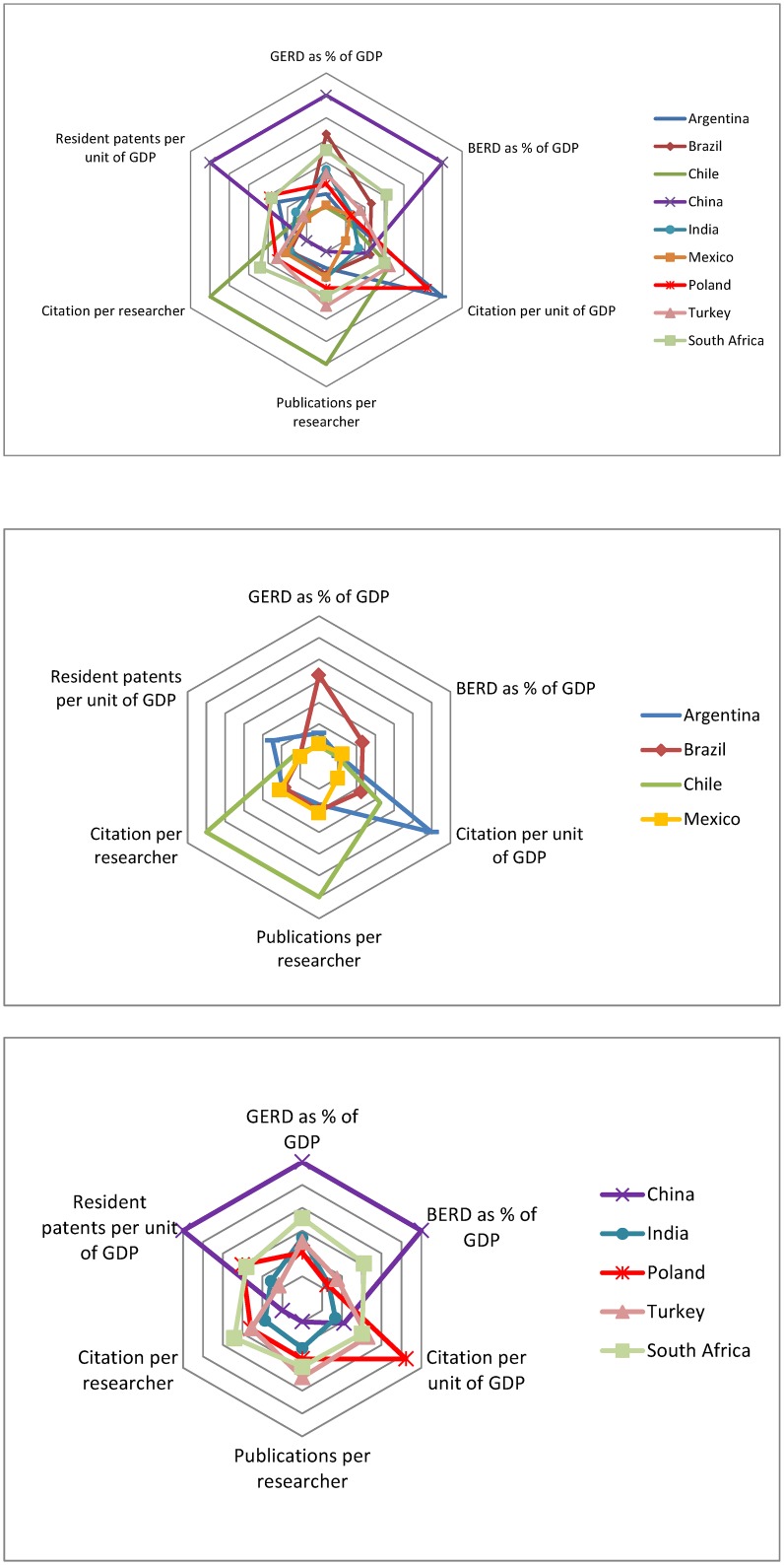
Input-Output for R&D across the baseline group. Sources: GERD, BERD and the number of full time equivalent (FTE) researchers data were obtained using the OECD Main Science and Technology Indicators (http://stats.oecd.org/Index.aspx?DataSetCode=MSTI_PUB). The number of publications and citations was obtained using the ISI National Science Indicators (Thomson Reuters, 2011). The GDP was obtained from the World Bank Indicators www.worldbank.org. Patents were obtained from WIPO, *Industrial Property Statistics—Aggregate Patent Data*, (2013).http://www.wipo.int/ipstats/en/statistics/patents/index.html.

Fig 2 presents a footprint graph equivalent to the one presented in Fig 1, but only for the set of developing countries of interest. The first observation is the important heterogeneity that it displays, especially if compared to what was found for the developed world [3]. All the countries have an asymmetric footprint, ranking high in certain dimensions and low in others. This heterogeneity is also present within each individual indicator. A major variation in the level of involvement of the business community in R&D among these nations can easily be noted, with China, South Africa and Brazil leading the group, and Turkey also above the average. Likewise, we can see that patenting activities also vary a lot among these nations, with China as the clear leader. The area where the difference between top and lower performers appears to be smaller is citation per unit of GDP.

The behavior of individual countries is also remarkable. First, Poland and Latin American countries, excluding Brazil, all have a modest to low level of investment in R&D, both overall, and in their private dimension. Nevertheless, they have an average to superior performance in terms of scientific productivity, above many of the other nations that are committing relatively more resources to R&D. Chile is a particularly interesting case, because it outperforms all others in terms of citations per researcher and publications per researcher. It has been pointed out that Chile’s scientific performance is related to its excellent collaboration with European researchers, in particular in research projects developed in the European Southern Observatory located in Chilean territory [9]. Even so, this suggests that the country has a small but highly productive S&T system, which could quickly grow in overall impact if the investments in R&D were to increase and productivity maintained.

China, Brazil and South Africa provide a good contrast to the nations noted in the previous paragraph. They are clear leaders of the group in terms of investment in R&D, both in relative and absolute terms, but their systems appear to have limited productivity, at least when considering these standardized international metrics. Interestingly, if we combine Figs 1 and 2 with figure 5 in [3], we can see that Asian countries (Japan, South Korea and China), have in general a low productivity in terms of publications and citations per researcher. The low productivity of these nations could represent a bias towards national journals, or at least a focus on outputs that are not usually considered in international scientific circles. In addition, it is possible that there is a trade off in the type of research of the various systems, so that a greater industrial focus may necessarily mean more patents at the expense of publications/citations [10].

India and Poland appear to have the most consistent pattern in terms of the levels of investment and scientific productivity. However, they diverge with respect to the citations per unit of GDP, where both countries outperform the rest of the group by far. This suggests that these two countries are much more active participants in international science and invention than what we might have expected, given the size of their economies. South Africa is also worth noting, as it seems to be the most balanced nation, with high investments in R&D, and a corresponding high performance in terms of scientific productivity and citation intensity, as well as relatively high level of invention activity as measured by patents.

### Research footprint

**Fig 3:**
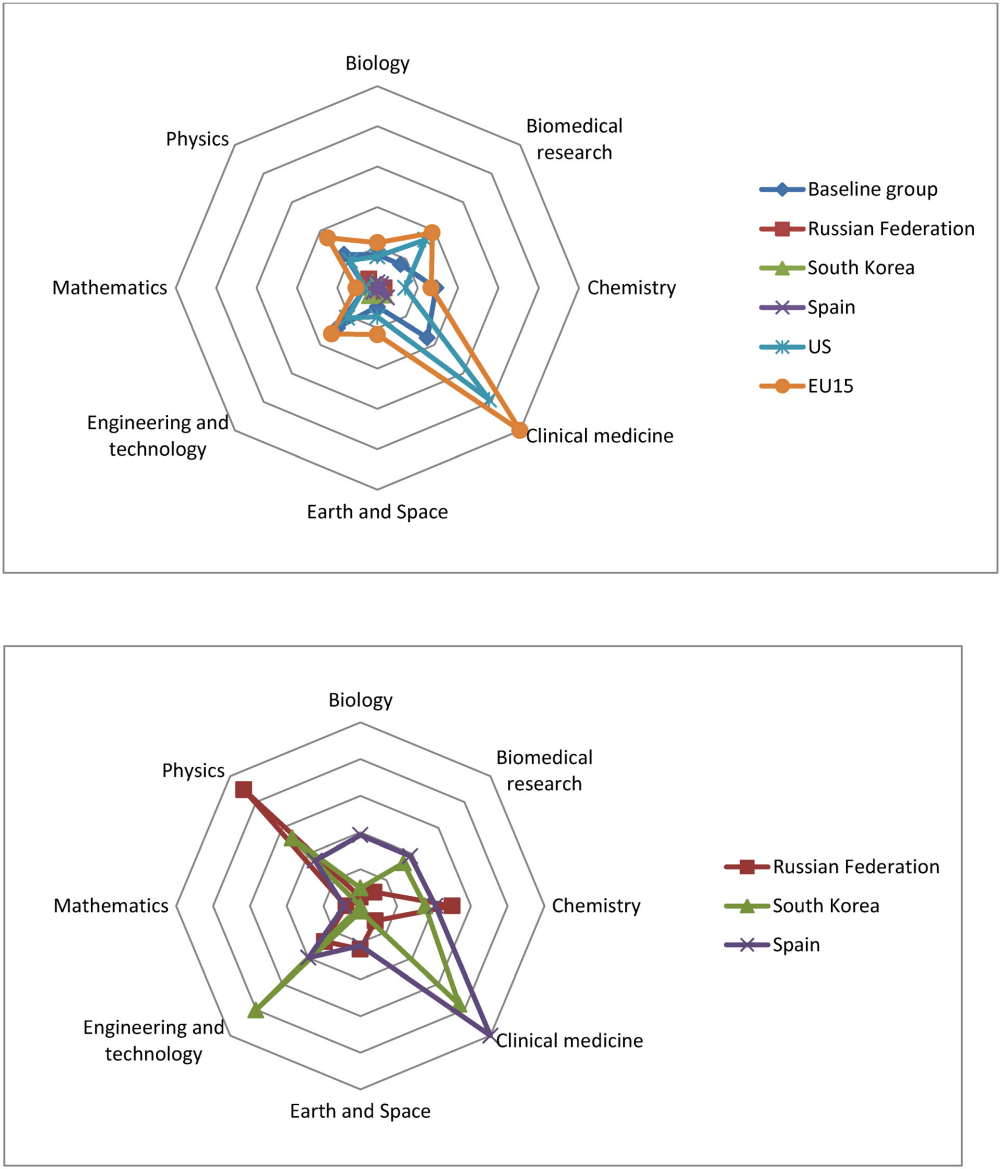
Research footprint baseline group vs. developed economies. Source: Thomson Reuters, Web of Science (Science Citation Index Expanded), compiled by UNESCO, (UNESCO, 2010).

As suggested by [11] new pictures are needed for interpreting research performance. Countries have established different priorities in R&D that are reflected in their outcomes. Fig 3 shows the normalized number of publications in the Science Citation Index Expanded in 2008 [12] for the baseline group and the benchmark develop nations. The analysis of publications in the Social Citation Index is not considered in this paper since there is some evidence [13] that in Social and Humanities an important number of research products are publish in the form of books and book chapters; and in local journals.

By far, the majority of the papers published are in Clinical Medicine in all countries but the Russian Federation, even for the baseline group. The chart confirms the findings of [14–15]. Clearly, R&D in the US and EU-15 has focused on the “Western Model” [14] where the biomedical research prevails. The Russian Federation continues focusing in chemistry and physics, and South Korea has focused more in engineering. The baseline group (as an added group) seems following a similar pattern than the “Western Model”, although the biomedical research is clearly smaller, probably because this research is more expensive and the participation of the private sector has shown to be imperative [15].

**Fig 4:**
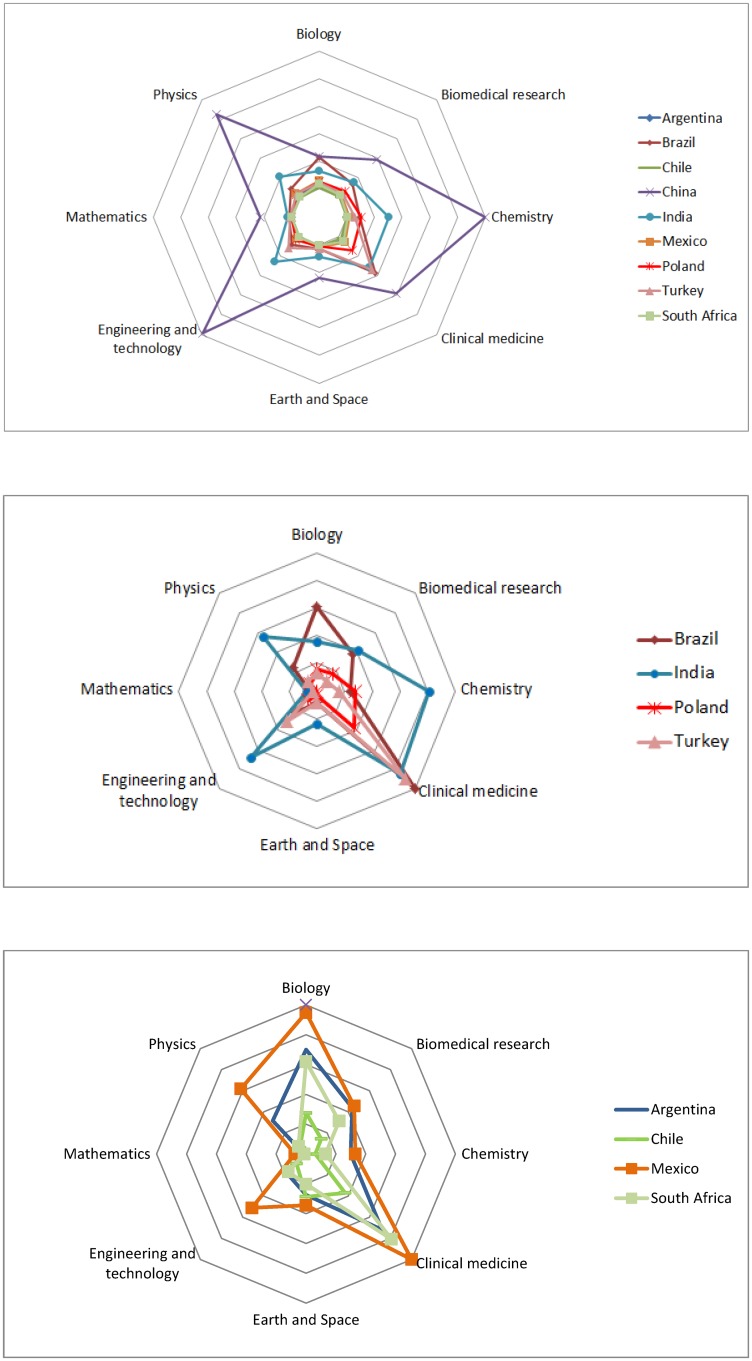
Research footprint for the baseline group. Source: Thomson Reuters, Web of Science (Science Citation Index Expanded), compiled by UNESCO, (UNESCO, 2010).

However, the differences among the countries of the baseline group are enormous. Fig 4 shows a footprint equivalent to the one presented in Fig 3 but only for the set of developing countries of interest. Clearly, China has focus its R&D on what [15] called Factor 2, with a high emphasis in Chemistry, Physics and Engineering, and a focus on innovation that coincides with its high participation of private investment in R&D and the elevated number of resident patents by unit of GDP (showed in Fig 2). India, on the other side, represents a mix of Factor 1 and Factor 2 countries, as China, India has a strong R&D in Chemistry, Physics and Engineering (Factor 2) but also in Clinical Medicine (Factor 1). However, India has a relatively low number of resident patents and the contribution of the business sector is modest, even though its strong domestic pharmaceutical industry.

The Latin-American countries considered in the sample, and South Africa are also a mix of Factor 3 and Factor 1 countries. Their R&D is strong in Biology and agricultural sciences (Factor 3) but also they are closing the gap with more research in Clinical Medicine and Biomedical research.

Finally, Poland and Turkey are Factor 1 countries and their R&D is more focused on Clinical Medicine, especially Turkey.

It is important to note that the number and characteristics of publications varies a lot among different areas of knowledge. In physics it is not uncommon to find publications with hundreds of authors; on the other side, mathematicians tend to publish in small teams. Other example is in clinical medicine, where one can find publications of 1 or 2 pages that show the results of clinical test, on the other extreme there are other areas where the papers are very large.

### International Collaboration

The increasing contribution of the baseline group to world S&T in outputs is closely related to how much researchers collaborate with other researchers in other countries. As stated by [16].

“the number of countries represented in the author list increases, articles are more likely to be published in journals with higher impact factors and accrue more citations than peer publications which have fewer countries represented”.

**Fig 5:**
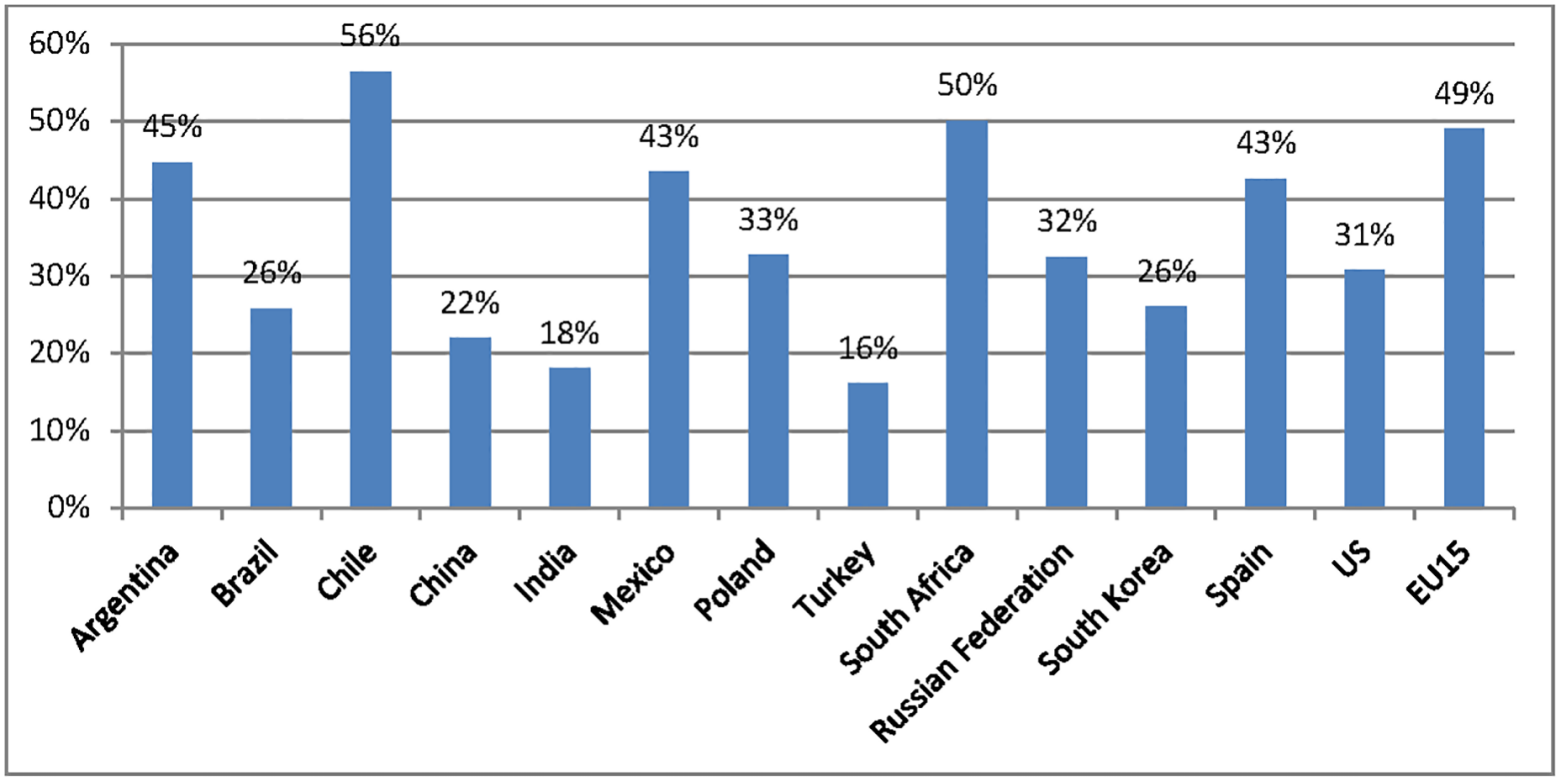
Percentage of Publications in International Collaboration. Source: Thomson Reuters, Web of Science (Science Citation Index Expanded), compiled by UNESCO, (UNESCO, 2010).

Thus, our measures of publications and citations have an international collaboration bias. Fig 5 shows the percentage of scientific publications in international collaboration in the Science Citation Index Expanded produced by Thomson Reuters [12]. As can be seen, Chile and South Africa are the countries with the highest international collaboration, and are also among the most productive, in terms of publications and citations per researcher. However, the variation among the baseline group is significant and, in some sense proves that the reduction in the science gap, between this set of developing countries and the developed countries, is not explained only by the international collaboration. Turkey is the second most productive country, in terms of publications per researcher; however, it has the least international collaboration, showing that there are other factors that also have help to reduce the science gap.

## Discussion and Conclusions

Since the early nineties, nine of the most dynamic developing economies have reduced the gap with the more developed world, both in terms of R&D investment and scientific impact. The results of this research shows that the baseline group of developing countries grew in absolute terms at almost double the rate of the world investment on R&D. Even more, the baseline group grew at more than three times the rate of world scientific output, measure as the number of publications. China in particular had a very strong evolution, becoming an important contender in the world S&T arena. It has been documented the critical role of universities in the building of China's national innovative capacity over the last years [17]. But the national benchmarks of inputs and outcomes reveals that this “catching up” hides important diversity in these S&T systems, not only in terms of how much they are committing to R&D given the relative size of their economies, but especially of what they are able to generate in terms of impacts and outputs. For example, Poland´s citation share was 0.73% in the 2005–2009 period, which almost double its GERD share that was 0.3% in 2009. This shows that it is a higher impact contributor in comparison to what it is investing. Argentina, Chile and Spain are also among the higher impact contributors.

It is clear that size, either of the nation or the magnitude of the investment, is not the key driver of efficiency. The differences seem to be rooted in the specific nature of the system and the institutions in each nation, which need to be better understood and compared in their specificities [18]. Chile appears to have been able to reach high levels of scientific productivity, despite its low level of R&D expenditures, by taking advantage of international collaborations and the European Southern Observatory [9]. Besides, it has the highest percentage of publications in international collaboration (56%) among the baseline group. Argentina and Spain are also countries with high international collaboration, which makes one presume that their high level of efficiency is due to that. However, Poland does not have a high rate of international collaboration (33%) and is the one that shows the highest efficiency, in terms of inputs/impacts. This suggests that a careful analysis of the research environment in these nations might help us understand the mechanisms that allow nations to reach such performance, aiming to draw lessons for other countries. Effective S&T policies require a careful understanding of the specific realities of each nation, as well as an attentive assessment and active search for best practices, to this purpose, it is highly recommended to use primary sources of information.

On the other side, the analysis in this paper shows that there are other nations that invest more, but achieve inferior levels of relative impact such as Brazil, China and India. All these countries have relatively low levels of international collaboration, 26%, 22% and 18%, respectively, which could be one explanation of their comparatively low level of citations. Another explanation could be that all these countries are non-English speaking countries, and there are studies that show evidence that China or Spain lose up to 13% and 8%, respectively, of their citations in ISI databases [19], because the citations are calculated by matching procedures that are fully automated, so misspellings are not taken into account. Another motive is that ISI performance indicators consider only journals included in Thomson Reuters databases, so that the count of citations of local journals, books, proceedings, among others, are not considered [8]. It could be expected that South Korea and the Russian Federation could also be in this situation. Finally, another explanation is that all countries included in this analysis have different knowledge footprints, and the number of publications and citations, as well as the investment that each discipline requires, varies a lot among areas of knowledge [8].

Another important conclusion of this paper is that the more developed countries outperform the baseline group in almost all dimensions but publications; and two of the most significant gaps are in the business side of the expenditures (BERD as % of GDP) and patents. As was mentioned before, this could be a consequence that developing countries’ S&T institutions were inspired by what is known as the “linear model”, assuming that good-quality basic research could produce, eventually, applied research.

It is important to stress that the analysis that has been done in this paper only shows selected S&T indicators, however, by looking at the figures along with exploring the drivers of private investments in R&D in nations such as Brazil, China and Poland might yield new ideas or strategies for the rest, as they strive to increase the involvement of their own business sector in S&T activities.

Finally, the figures presented in this paper show the great heterogeneity of these countries. Notwithstanding we believe that the analysis exhibits how deepening our understanding of the S&T system in these nations, especially by contrasting similarities and differences among them, can provide significant learning opportunities, helping to foster sounder S&T policies that can help these nations make the most of their R&D investment. It is important to stress that the measures of inputs and outputs shown in this paper have important limitations since they do not capture the complexity of the R&D process. There are other inputs more than GERD and BERD that influences the outputs, such as S&T infrastructure, the level of education of the population, competitiveness indicators, the incline to international collaboration and the priorities of certain areas of knowledges. There are areas of knowledge that require more resources than others [15]. On the other side, there are also a lot of outputs that are crucial and this study does not take into account [20]. Moreover, it has been documented that publications, citations and patents are poor proxies of the outputs of S&T since to capture the essence of good science, evaluators of scientific activity should combine forces to create an open, sound and consistent system for measuring all the activities that make up academic productivity [13, 20–25]. In this paper, we only consider publications and citations reported in ISI databases, and even though they are a valuable tool in policy studies addressing general issues regarding academic systems since they are objective measurements of the diffusion and impact of research, and allow us to determine the geographic origin of research and detect growth or erosion of countries´ scientific impact; they have important limitations, such as they do not take into account books, proceedings, local journals, etc., besides the count of citations does not consider misspellings [26].

Another important caveat of this paper is that it is based on S&T indicators reported by the nations to international agencies, and even though the multiple efforts of agencies like UNESCO and OECD to standardize indicators and make them comparable across nations, the methodologies used could lead to different conclusions, so the indicators used in this study could be having dissimilar meaning or calculated using deviating methodologies [21, 27]. Thus, the conclusions of this paper depend on the accuracy of the source data that was used.

The other aspect that is crucial to stress is that we are attributing inputs and outputs/impacts to each country. However, this is no valid anymore. Nowadays, R&D has no frontiers, and both the creation and the spillovers of knowledge are increasingly becoming global [27].

## Supporting Information

S1 Table Summary statistics. (DOCX)
