# Supplementary material for: The Scientific Impact of Developing Nations
Source: PLoS One. 2016 Mar 29;11(3):e0151328. doi: 10.1371/journal.pone.0151328 (PMC4811426; doi:10.1371/journal.pone.0151328)
Supplement: S1 Table — (DOCX) [file pone.0151328.s001.docx]

**S1 Table: Summary statistics**

*GDP: Gross Domestic Product; GERD: Gross Domestic Expenditure on R&D; BERD: Business Expenditure on R&D
